# P-1871. Scalability Metrics and Effort Requirements for a Long-Acting CAB/RPV Program

**DOI:** 10.1093/ofid/ofaf695.2040

**Published:** 2026-01-11

**Authors:** Jennifer O’Neill, Maureen Kubat, Shawnalyn Sunagawa, Sara H Bares, Nada Fadul, Jennifer M Davis, Josh Lechner, Josh Havens

**Affiliations:** University of Nebraska Medical Center, Omaha, Nebraska; University of Nebraska Medical Center, Omaha, Nebraska; University of Nebraska Medical Center, Omaha, Nebraska; University of Nebraska Medical Center, Omaha, Nebraska; University of Nebraska Medical Center, Omaha, Nebraska; University of Nebraska Medical Center, Omaha, Nebraska; University of Nebraska Medical Center, Omaha, Nebraska; University of Nebraska Medical Center, Omaha, Nebraska

## Abstract

**Background:**

As long-acting injectable (LAI) therapies for HIV like cabotegravir and rilpivirine (CAB/RPV) gain wider adoption, understanding the operational demands of real-world implementation is essential. This study describes the effort and resources required to support a LAI CAB/RPV program.

**Methods:**

We conducted a retrospective time allocation analysis of the CAB/RPV LAI program at the University of Nebraska Medical Center. Staff effort (e.g., program start-up, coordination, benefits, clinical) was estimated from May 2022 to December 2024 under a buy-and-bill model. Before May 2022, the site participated as a CAB/RPV investigational site. The primary outcome was estimated staff effort (FTE), with roles categorized for analysis. Scalability metrics were derived from FTE estimates. Secondary outcomes included injection metrics (e.g., delayed/missed doses) and virologic failure. Data were summarized descriptively.

**Results:**

Within the study period, 113 patients received 884 CAB/RPV injections (Table 1); total number of discontinuations was 8 (7%). Mean age was 46 years; 79% cisgender men, 18% Black, 13% Hispanic, and 11% Medicaid. Effort requirement estimations included an LAI program start-up period (i.e. protocol development, workflow, etc.) of 0.24 FTE (269 hours) and scaling from 1.0 FTE (2080 hours) to 2.25 FTE (4680 hours) from May 2022 through 2024 (Figure 1). The greatest cumulative effort by task included LAI program coordination (3680 hours), LAI program iteration (1456 hours), and patient outreach (888 hours) (Figure 2). Mean overall CAB/RPV patients managed/FTE, new CAB/RPV patients managed/FTE, and injections/FTE were 39 patients, 25 patients, and 166 injections, respectively (Table 1). There were 3 patients who required oral bridging, 3 delayed injections (median time beyond injection window, 5 days; range, 3-7 days), and no missed injections. To date, no confirmed virologic failures have been observed.

**Conclusion:**

Implementation of an LAI CAB/RPV program requires substantial personnel effort, particularly for coordination and patient support tasks. Adequate staffing is critical for successful scale-up of LAI programs. Despite operational demands, clinical and implementation outcomes were favorable, supporting feasibility in real-world settings.

**Disclosures:**

Nada Fadul, MD, ViiV Healthcare: Advisor/Consultant|ViiV Healthcare: Grant/Research Support Jennifer M. Davis, MD, Viiv: Grant/Research Support Josh Havens, PharmD, Gilead Sciences: Grant/Research Support|Merck: Advisor/Consultant|ViiV Healthcare: Advisor/Consultant

